# Sensitivity and specificity for African horse sickness antibodies detection using monovalent and polyvalent vaccine antigen-based dot blotting

**DOI:** 10.14202/vetworld.2022.2760-2763

**Published:** 2022-12-05

**Authors:** Machimaporn Taesuji, Khate Rattanamas, Usakorn Kulthonggate, Thanongsak Mamom, Sakchai Ruenphet

**Affiliations:** 1 Master of Science Program in Animal Biotechnology, Faculty of Veterinary Medicine, Mahanakorn University of Technology, Bangkok, Thailand; 2 Clinic for Horse, Faculty of Veterinary Medicine, Mahanakorn University of Technology, Bangkok, Thailand; 3 Department of Pathology, Faculty of Veterinary Medicine, Mahanakorn University of Technology, Bangkok, Thailand; 4 Department of Immunology and Virology, Faculty of Veterinary Medicine, Mahanakorn University of Technology, Bangkok, Thailand

**Keywords:** African horse sickness, blocking enzyme-linked immunosorbent assay, dot blotting, sensitivity, specificity

## Abstract

**Background and Aim:**

The immune responses of animals infected with African horse sickness (AHS) virus are determined by enzyme-linked immunosorbent assay (ELISA), complement fixation, and virus neutralization test. During the outbreaks of AHS in Thailand, the immune response after vaccination has been monitored using commercial test kits such as blocking ELISA, which are expensive imported products unavailable commercially in Thailand. This study aimed to assess the sensitivity and specificity of anti-AHS virus antibodies using dot blotting based on monovalent and polyvalent strains of live attenuated AHS vaccine.

**Materials and Methods:**

A total of 186 horse sera, namely, 93 AHS-unvaccinated samples and 93 AHS-vaccinated samples, were used in this study. All sera underwent antibodies detection using commercial blocking ELISA and in-house dot blotting based on monovalent and polyvalent strains of live attenuated AHS vaccine. The numbers of true positive, false positive, true negative, and false negative results in the dot blotting were compared with those in blocking ELISA and the sensitivity and specificity of dot blotting were assessed.

**Results:**

For the monovalent antigen, there were 78, 19, 74, and 15 true positive, false positive, true negative, and false negative results, respectively, while for the polyvalent antigen, the corresponding numbers were 84, 34, 58, and 9. Meanwhile, the diagnostic sensitivity and specificity for monovalent antigen were 83.87% and 79.57%, respectively, but 90.32% and 62.37% for polyvalent antigen.

**Conclusion:**

Dot blotting for AHS antibodies detection using vaccine antigen showed high sensitivity and rather a high specificity compared with the findings with the commercial ELISA test kit. In countries where commercial ELISA test kits are not available and when the size of a serum sample is small, dot blotting could become a good alternative test given its advantages, including its simplicity, rapidity, and convenience. To the best of our knowledge, these findings are the first report on the use of dot blotting for detecting AHS antibodies in horses. In conclusion, monovalent antigen-based dot blotting could be used as a reliable alternative serodiagnostic test for monitoring AHS humoral immune response, especially in vaccinated horses.

## Introduction

African horse sickness (AHS) is a vector-borne, non-contagious viral disease caused by the AHS virus (AHSV), which affects all Equidae species. The disease causes high mortality in affected horse populations and is included in the World Organization for Animal (OIE) listed diseases. African horse sickness virus is an RNA virus of the family Reoviridae, genus *Orbivirus*. It has been classified into nine serotypes based on viral capsid protein 2 [1, 2]. Although AHS is an endemic disease in Africa, in March 2020, ASHV serotype 1 [3, 4] emerged in northeast Thailand [3, 5, 6]. Generally, AHS causes economic losses not only through direct mortality of horses but also through restrictions on movement imposed to reduce spread, culling of infected animals, and the implementation of *Culicoides* control and horse vaccination strategies for disease control and prevention [7].

In countries where AHS is endemic or emerging, AHS prevention is highly dependent on vaccination, which can clearly protect against this devastating disease. Standard post-vaccination serological tests for monitoring immune responses are enzyme-linked immunosorbent assay (ELISA), complement fixation, and virus neutralization [8]. Commercial ready-made test kits, such as blocking ELISA are available in Africa and Europe in particular, but they are expensive and unavailable in Thailand.

This study aimed to assess the sensitivity and specificity of anti-AHSV antibodies using dot blotting based on monovalent and polyvalent strains of live attenuated AHSV.

## Materials and Methods

### Ethical approval

Guidelines used for the care and use of animals were approved by the Animal Research Ethics Committee, Faculty of Veterinary Medicine, Mahanakorn University of Technology, Thailand, approval number ACUC-MUT-2021/003.

### Study period and location

This study was conducted from October 2020 to May 2022 at the biosecurity level-2 facilities of the Virology and Molecular Diagnostic Laboratory, Faculty of Veterinary Medicine, Mahanakorn University of Technology, Bangkok, Thailand.

### Horses and serum preparation

The serum samples used in this study were divided into two groups. In the first group, 93 archived horse sera at the Virology Section of Mahanakorn Veterinary Diagnostic Center, Faculty of Veterinary Medicine, Mahanakorn University of Technology, Thailand, were used as unvaccinated control sera. These sera were collected before the AHS outbreak in Thailand for other purposes. The second group of samples consisted of 93 horse sera collected at least 30 days after AHS vaccination. These sera were provided by First Livestock and Agriculture Division, Veterinary and Remount Department, Royal Thai Army, Kanchanaburi Province, Sap Takhian Cowboy City and KC Horse Farm, Nakhon Sawan Province, Thailand. The sera were stored at −30°C before testing.

### Blocking ELISA

The AHS antibodies’ titers of the 186 serum samples were tested using a commercial ELISA kit (Ingezim AHSV Compac Plus^®^, Ingenasa, Madrid, Spain). Briefly, this kit is based on blocking ELISA, involving a reaction between the recombinant VP7 protein adsorbed on the ELISA plate and a peroxidase-conjugated AHS-VP7-specific monoclonal antibodies. This study determined the blocking percentage (BP) of each serum sample in accordance with the manufacturer’s recommendations. The BP value is calculated using the following formula:


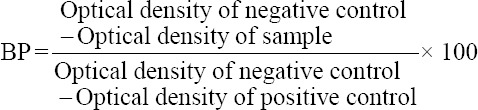


BP value of <45% is considered to reflect negative results, while one of ≥50% reflects positive results. However, BP value between 45% and 49% is considered doubtful, and they must be retested, if the result is the same, another extraction must be made and tested 2 weeks later.

### Dot blotting

#### Antigens

African horse sickness virus antigens from the live attenuated AHS monovalent and polyvalent vaccines (Onderstepoort biological product, Pretoria, South Africa) were used in this study. These vaccines were dissolved in accordance with the manufacturer’s recommendations. The dissolved vaccine virus was aliquoted and kept at −80°C until testing.

#### Nitrocellulose membrane

A nitrocellulose membrane with a pore size of 0.45 μm (Boster Biological Technology, California, USA) was used for dot blotting in this study.

#### Dot blotting procedure

The nitrocellulose membrane was cut into a small rectangular piece of 2 × 1 cm. Five microliters of each antigen was coated on the membrane and kept in a Class II biosafety cabinet for drying. Then, the coating membrane was blocked using 5% skim milk in phosphate-buffered saline (PBS) and incubated overnight on a shaker at room temperature. Subsequently, the coating membrane was washed with a washing solution (0.05% Tween20 with 5% skim milk in PBS) 3 times for 5 min each. The coated membrane was placed in a plastic pouch and a test serum sample at a dilution of 1:5 was added, followed by incubation for 1 h in a 37°C incubator. After three washes with washing solution, the membrane was immersed with 1:10,000 anti-horse IgG antibodies conjugated with peroxidase (Rockland Immunochemicals Inc., Pennsylvania, USA) diluted with 5% skim milk, placed in a plastic pouch, and incubated for 1 h in a 37°C incubator. The membrane was then washed three times with 5% skim milk in PBS. Finally, TMB substrate (SureBlue Reserve™ TMB 1-component Microwell peroxidase substrate, LGC Seracare, Massachusetts, USA) was dropped on the sample and the result was read immediately and recorded.

### Evaluation of sensitivity and specificity

The dot blotting results were compared with the standard blocking ELISA test results. If these sets of results were correlated, this was interpreted as either true positive or true negative, whereas the uncorrelated results were interpreted as either false negative or false positive. The numbers of true positive, false positive, true negative, and false negative results of dot blotting compared with blocking ELISA were evaluated and the sensitivity and specificity of dot blotting were determined as follows:


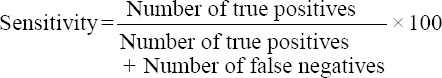



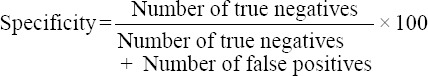


## Results

**Figure-1:**
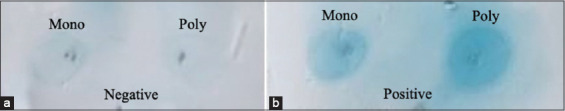
The result of dot blotting using monovalent and polyvalent antigens of live attenuated African horse sickness virus vaccine. (a) Negative result; (b) Positive result; Mono=Monovalent antigen; Poly=Polyvalent antigen.

Dot blotting for the detection of AHS antibodies using monovalent and polyvalent vaccine antigens showed both negative and positive results, as demonstrated in Figure-1. Overall, for the monovalent antigen, there were 78, 19, 74, and 15 true positive, false positive, true negative, and false negative results, respectively, while for the polyvalent antigen, the corresponding numbers were 84, 34, 58, and 9 (Table-1). For monovalent antigen, the detection sensitivity and specificity were 83.87% and 79.57%, respectively, while for polyvalent antigen, they were 90.32% and 62.37% (Table-2).

**Table-1:** The result of dot blotting using monovalent and polyvalent antigen of live attenuated African horse sickness virus vaccine compared to blocking ELISA.

Test	Dot blotting			

	Monovalent		Polyvalent	
	
	Positive	Negative	Positive	Negative
ELISA				
Positive	78	15	84	9
Negative	19	74	34	58

ELISA=Enzyme-linked immunosorbent assay

**Table-2:** The diagnostic sensitivity and specificity of dot blotting using monovalent and polyvalent antigen of live attenuated African horse sickness virus vaccine compared to blocking ELISA.

Antigen type	Monovalent (%)	Polyvalent (%)
Sensitivity	83.87	90.32
Specificity	79.57	62.37

ELISA=Enzyme-linked immunosorbent assay

## Discussion

In this study, negative sera were collected before the AHS outbreak in Thailand, and positive sera came from AHS-vaccinated horses. Overall, 100% of these serum samples were confirmed as negative and positive serum controls using a standard serological test, namely, blocking ELISA, which has been highlighted as the standard method for detecting AHS antibodies by several research groups, such as Durán-Ferrer *et al*. [9] and OIE [8]. In this study, the detection sensitivity of polyvalent antigen was high (90.32%), but the specificity was too low (62.37%) due to an abundance of false positives. This suggested that lack of purity of antigen in live attenuated vaccines might play an important role in non-specific or cross-immune reactions. For monovalent antigen, the detection sensitivity was high (83.87%), as was the specificity (79.57%). These results demonstrate that the specificity of monovalent antigen was higher than that of polyvalent antigen, although the sensitivity was lower. This study indicated that dot blotting based on monovalent antigen is far superior in terms of the specificity of detection.

Given that dot blotting and ELISA are based on the same principle, several research groups described and compared the results from these two tests, such as Yamchi *et al*. [10], Taher *et al*. [11], and Chatziprodromidou and Apostolou [12]. In this study, the level of sensitivity of dot blotting for AHS antibodies detection was higher than its specificity. These results are correlated with the findings in the study by Chatziprodromidou and Apostolou [12], who estimated the sensitivity and specificity of ELISA and dot blotting for detecting antibodies of *Neospora caninum* in dairy cows, as well as in the study by Yamchi *et al*. [10], who investigated the efficacy of in-house indirect ELISA and dot blotting for the sero-diagnosis of *Fasciola gigantica*. Interestingly, in this study, dot blotting for antibodies detection against AHS could be performed using vaccine antigen. It is thus feasible to perform this method in places where commercial ELISA test kits are unavailable, as well as in cases with a small sample size. To the best of the authors’ knowledge, this work is the first to report the detection of AHS antibodies in horses using dot blotting. However, this study did not include serum samples from infected horses, so further studies on sera from both naturally and experimentally infected horses are required.

For AHS vaccination using live attenuated vaccines, several concerns have to be considered, such as their possible reversion to virulence, transmission, reassortment with field AHSV strains, and the inability to differentiate infected from vaccinated animals [13–15]. All of the OIE member countries need to apply for recognition of freedom from AHS, which requires demonstration of the following: (i) no cases of infection with AHS virus for at least the last 2 years and (ii) no routine vaccination against AHS having been carried out during the last year [16]. Thailand has not had any reported ASH cases since September 2020 and ASH vaccinations were abandoned in 2021. For this reason, the Thai government is attempting to establish Thailand as an AHS-free country by applying to OIE in 2022. It is thus important to achieve a very detailed and thorough recording of the vaccination campaign, particularly the number of animals vaccinated versus the number of registered equids. However, some selected horses (e.g., young horses, pregnant horses, and sentinel horses) should be sampled and tested to check for viral infection in the vaccinated population [17]. The current study demonstrated that dot blotting is useful for detecting antibodies against AHS. However, false positive results might occur, so care should be taken when interpreting positive results from unvaccinated horses.

## Conclusion

Monovalent antigen-based dot blotting could be used as a reliable alternative serodiagnostic test for AHS monitoring, especially in vaccinated horses. Furthermore, dot blotting is more simple, rapid, and convenient than blocking ELISA and serum neutralization tests.

## Authors’ Contributions

MT, KR, UK, TM and SR: Study conception and design, conducted the experiments, and analyzed the data. SR: Sample preparation. MT, TM, and SR: Drafted the manuscript. All authors have read and approved the final manuscript.
